# The Probiotic Effectiveness in Preventing Experimental Colitis Is Correlated With Host Gut Microbiota

**DOI:** 10.3389/fmicb.2018.02675

**Published:** 2018-11-01

**Authors:** Sharmila Suwal, Qiong Wu, Wenli Liu, Qingya Liu, Hongxiang Sun, Ming Liang, Jing Gao, Bo Zhang, Yanbo Kou, Zhuanzhuan Liu, Yanxia Wei, Yugang Wang, Kuiyang Zheng

**Affiliations:** Jiangsu Key Laboratory of Immunity and Metabolism, Laboratory of Infection and Immunity, Department of Pathogenic Biology and Immunology, Xuzhou Medical University, Xuzhou, China

**Keywords:** probiotics, inflammatory bowel disease, microbiota, personalized medicine, biotherapy

## Abstract

Current evidence to support extensive use of probiotics in inflammatory bowel disease is limited and factors that contribute to the inconsistent effectiveness of clinical probiotic therapy are not completely known. Here, we used *Bifidobacterium longum* JDM 301 as a model probiotic to study potential factors that may influence the effect of probiotics in experimental colitis. We found that the effect of *B. longum* JDM 301 in tempering experimental colitis varied across individual mice even with the same genetic background. The probiotic efficacy was highly correlated with the host gut microbial community features. Consumption of a diet rich in fat could exacerbate mucosal injury-induced colitis but could not change the host responsiveness to *B. longum* JDM 301 treatment, suggesting of potential mechanistic differences between regulating colitis pathogenesis, and modulating probiotic efficacies by the gut microbiota. Together, our results suggest that personalized microbiome features may modify the probiotic therapeutic effect and support the idea of personalized probiotic medicine in inflammatory bowel disease.

## Introduction

Inflammatory bowel disease (IBD), including Crohn’s disease and ulcerative colitis, is a multi-factorial chronic inflammatory intestinal disorder with the highest prevalence in western countries (Leone et al., 2014; Ruemmele, 2016; Statovci et al., 2017). The etiology of IBD is currently incompletely understood. Both the host genetic factors and the gut microbiota contribute to the pathogenesis of IBD (Kaser et al., 2010). In experimental colitis models, it has been shown that the microbiota plays important roles (Taurog et al., 1994; Rath et al., 1996; Sellon et al., 1998). More importantly, changes of gut microbiota, i.e., dysbiosis, are frequently found in human IBD patients (Frank et al., 2007; Willing et al., 2009). However, whether dysbiosis is a cause or a consequence of IBD is still debatable. Western diet, which is high in fat, can induce dysbiosis and is considered as a risk factor for the development of IBD (Hildebrandt et al., 2009; Chapman-Kiddell et al., 2010). Strategies for manipulating microbiota composition and metabolism can potentially be utilized to treat gut inflammation, but many practical and theoretical challenges remain to be overcome.

Probiotics, which are defined as “live microorganisms which when administered in adequate amounts confer a health benefit on the host” (Hill et al., 2014), are thought to be able to actively modulate the gut microbiota and provide therapeutic benefits across a broad range of disorders including IBD (Martin et al., 2015; Jiang et al., 2016; Kim et al., 2017). Probiotics are in fact often recommended by physicians as adjunctive therapy to induction or maintenance of remission in IBD (Cheifetz et al., 2017). Despite their popularity, the current evidence to support extensive use of probiotics in IBD is limited. Results from clinical trials with probiotics in IBD are mixed, with some studies showing an improvement in maintenance or induction of remission while other trials have failed to show any beneficial effect (Derwa et al., 2017; Yamamoto et al., 2017). The reason behind the various outcomes of probiotic effectiveness in IBD is not clear.

Gut microbiota not only affects disease pathology, but also plays many other important roles, including preventing pathogen colonization (Hand, 2016), shaping the immune system (Round and Mazmanian, 2009; Macpherson et al., 2017), stimulating the production of gastrointestinal hormones (Saulnier et al., 2013), regulating brain behavior through the production of neuroactive substances, and fermentation of non-digestible carbohydrates producing short-chain fatty acids (SCFAs) (Dinan et al., 2013; Groen et al., 2018). Most recently, the microbiota is also emerging as a contributing factor to interindividual variability in disease (Kashyap et al., 2017). However, whether the gut microbiota contributes to the person-to-person differences in response to probiotic therapy remains largely unknown.

*B. longum* JDM 301 is a widely used commercial probiotic strain in China (Wei et al., 2010). Our previous study demonstrated that *B. longum* JDM 301 can prevent *Clostridium difficile* infection (CDI) in mice (Wei et al., 2018). In the present study, we used *B. longum* JDM 301 as a model probiotic to test factors that could potentially influence the therapeutic effect of probiotics in experimental colitis. Our data demonstrated an association of the gut microbiota to interindividual variability in probiotic biotherapeutic responses. Our results suggest that personalized strategies are needed for the success of probiotic biotherapies in IBD.

## Materials and Methods

### Animals

Five- to six-week-old male C57Bl/6J mice were purchased from Shanghai Laboratory Animal Research Center, Shanghai, or Beijing Vital River Laboratory Animal Technology Co., Ltd., Beijing. Animals were maintained under specific pathogen-free (SPF) conditions with a 12-h light phase and 12-h dark phase and had free access to diet and drinking water. All animal experiments were done following the guidelines of the National Laboratory Animal Ethics Committee of China and were approved by the Animal Care and Use Committee of Xuzhou Medical University (Permit Number 2013-AN-65).

### Diet

Mice were fed with standard chow diet for the first week of arrival to the laboratory. Then the mice were randomly divided into two groups. One group of mice was fed with standard chow diet (ND) and the other group of mice was fed with high-fat diet (HFD), 60% energy from fat (Ke Ao Xie Li Co., Ltd., Beijing, China). HFD feeding was continued for a total of 6 weeks.

### Colitis Induction

Colitis was induced using dextran sulfate sodium (DSS) (Molecular Weight = 36,000∼50,000) (MP Biomedicals, Santa Ana, CA, United States). For mice in the ND group (5–8 mice), DSS treatment was started when the mice became 8 weeks of age. Whereas, for mice in the HFD group (5–8 mice), acute colitis was induced with DSS after 6 weeks of HFD feeding. Drinking water of both groups was replaced with 3% DSS (w/v) for 7 days during DSS challenge, followed by 3 days of recovery period during which sterilized drinking water was supplied. The control group (3–5 mice) fed with either ND or HFD received only sterilized drinking water throughout the experiment.

### Preparation of *B. longum* JDM 301 for Inoculation to Mice

*B. longum* JDM 301 was originally isolated from a commercial probiotic product from China (Wei et al., 2010). The frozen glycerol stock of *B. longum* JDM 301 was thawed and plated on MRS agar plate. The plate was incubated overnight at 37°C in an anaerobic incubator (Whitley Workstation DG250; Frederick, MD, United States) containing an atmosphere of 10% hydrogen, 10% carbon dioxide, and 80% nitrogen. The next day, a single bacterial colony was inoculated in a tube containing 3–5 ml of MRS broth and was incubated anaerobically for 16–24 h. The concentration of *B. longum* JDM 301 was evaluated according to OD600 and adjusted to 1 × 10^9^ colony forming unit (cfu) per 100 μl in sterile 1 × PBS. One week prior to DSS challenge, mice in appropriate groups received altogether three doses of 1 × 10^9^ cfu *B. longum* JDM 301 on three alternate days by oral gavage.

### Fecal Material Transplantation

Eight-week-old male C57Bl/6 mice maintained under SPF conditions were treated with a broad-spectrum antibiotic cocktail containing ampicillin (1 g/l), metronidazole (1 g/l), neomycin (1 g/l), and vancomycin (0.5 g/l) in their drinking water for 1 week. For fecal transplantation, fecal samples were suspended in sterile 1xPBS (10 mg/100 μl) and homogenized using Bio-Gen PRO200 Homogenizer (PRO Scientific Inc.). Homogenized feces were filtered through a 40 μm nylon cell strainer. Each antibiotic-treated mouse was orally gavaged with 200 μl of the filtrate daily for five consecutive days.

### Colonic Tissue Collection and Processing

The colonic tissues were collected and fixed in 4% (w/v) paraformaldehyde (pH 7.0), dehydrated by increasing concentrations of ethanol, and embedded in paraffin for histological studies. For histological grading three different parameters were considered, severity of inflammation (based on polymorphonuclear neutrophil infiltration; 0–3: none, slight, moderate, and severe), depth of injury (0–3: none, mucosal, mucosal and submucosal, and transmural), and crypt damage (0–4: none, basal one-third damaged, basal two-thirds damaged, only surface epithelium intact, entire crypt, and epithelium lost). The histological score for each mouse was a sum of the score of neutrophil infiltration, depth of injury and crypt damage.

### Gut Microbiota Analysis

Fresh fecal pellets were collected in a clean sterile Eppendorf tube, immediately frozen into liquid nitrogen, and then stored at -80°C. DNA was isolated using the E.Z.N.A. Stool DNA Kit (Omega Bio-Tek) according to the manufacturer’s instructions. Fecal DNA samples were amplified by PCR using barcoded primer pairs targeting the V3-V4 region of bacterial 16S rRNA gene using 341F/R806 primer sets. The PCR reaction mixture (20 μl) contained 10 ng genomic DNA, 0.25 μM of each primer and one-unit TransStart Fastpfu DNA Polymerase (TransGen, Beijing, China). The PCR reaction conditions for amplification of DNA were as follows: initial denaturation at 95°C for 3 min, followed by 27 cycles of denaturation at 95°C for 30 s, annealing at 55°C for 30 s and extension at 72°C for 45 s, and final ending at 72°C for 10 min. The PCR products were subsequently pooled and cleaned by agarose gel purification. PCR amplicons were then sequenced using a paired-end, 2 × 250-bp cycle run on an Illumina MiSeq sequencing system. Bioinformatic analysis was done by Vazyme Biotech Co., Ltd., Nanjing, China using the QIIME analysis pipeline. In brief, the paired-end reads were joined together after demultiplexing, fasta quality files and a mapping file indicating the barcode sequence corresponding to each sample were used as inputs, reads were split by samples according to the barcode, a taxonomical classification was performed using the RDP-classifier, and an OTU table was created. Closed-reference OTU mapping was employed using the Greengenes database. Sequences sharing 97% nucleotide sequence identity were binned into the same OTUs. Bacterial taxa summarization and rarefaction analyses of microbial diversity or compositional differences (dissimilarity value indicated by Unweighted UniFrac Distance) were then calculated and PCoA plots indicating compositional difference were generated accordingly with the Vegan package in R software.

### Data Availability

The 16S rRNA sequencing data have been deposited in the NCBI SRA database. The accession number is SRP149682.

### Statistical Analysis

The data are shown as mean values ± standard error of the mean (SEM). Differences between multiple groups were compared using one-way ANOVA with *post hoc* Turkey’s multiple comparison test and two-way ANOVA with *post hoc* Bonferroni posttests. A Student’s *t*-test was used for comparisons between two groups. Mantel-Cox test was used for survival analysis. Wilcoxon Signed Rank Test and Kruskal-Wallis (KW) sum-rank test were used as significance test in microbiota analysis. A *P*-value < 0.05 was considered significant.

## Results

### The Effectiveness of the Probiotic *B. longum* JDM 301 in Colitis Is Correlated With Host Microbiota

**FIGURE 1:**
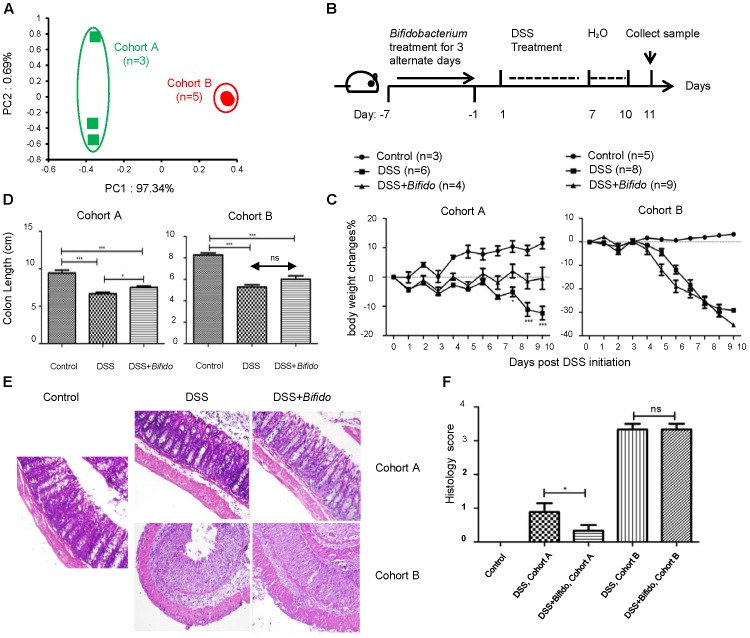
The effectiveness of the probiotic *B. longum* JDM 301 in colitis is correlated with host microbiota. **(A)** PCoA analysis based on OTU abundance of each mouse via fecal bacterial 16S rRNA sequencing. Each symbol represents one individual mouse. The fecal samples were collected from mice fed on a normal diet (ND) before probiotic and DSS treatment. **(B)** Schematic diagram of experimental design. **(C)** The body weight changes during DSS treatment. **(D)** Mean colon length in cm. Colons were collected on day 11 post DSS initiation. **(E)** Representative images of H&E stained distal colon tissues from indicated mice (magnification: 200x). **(F)** Histologic scores. All data are given as means ± SEMs. ns, no statistical significance. ^∗^*P* < 0.05; ^∗∗∗^*P* < 0.001.

The effectiveness of probiotics in IBD remains unclear (Derwa et al., 2017; Yamamoto et al., 2017). We posited that one of the reasons might be related to the highly varied host microbiota. To test this hypothesis, we actively looked for C57Bl/6 wild-type (WT) mice that harbored different microbiota by screening their fecal bacterial 16S rRNA sequences. We found two cohorts of mice, which were purchased from the same vendor in a different time, turned out to have significant differences in their gut microbial communities based upon unweighted UniFrac principal coordinate analysis (PCoA) (Figure 1A).

We used *B. longum* JDM 301 (Wei et al., 2010), a widely used commercial probiotic strain in China, to test whether there is any effect of this specific probiotic in tempering colitis established in mice. Each mouse was orally gavaged with *B. longum* JDM 301 (1 × 10^9^ cfu in 100 μl PBS/mouse) for 3 alternate days, colitis was then induced by replacing drinking water with 3% DSS for another 7 consecutive days and then switched back to normal drinking water (Figure 1B).

Before DSS treatment, the mean initial body weight of mice belonging to both cohorts was similar (around 23.5–23.8 g). At the end of experimental period (i.e., 10 days post-infection), in *B. longum* JDM 301 non-treated colitic mice, the loss in body weight was more pronounced in cohort B mice (around 30%) compared to cohort A mice (around 15%) (Figure 1C). Furthermore, all mice in cohort A survived the intestinal injury-induced wasting disease; while 7 out of 8 mice in cohort B died within the experimental period.

To determine whether the different colitis sensitivity of the two cohorts of mice was related to their microbiota, we transferred the fecal contents from cohort A or B mice to the third batch of mice that were pretreated with antibiotics. We then challenged the third batch of mice with DSS. The mice receiving feces from cohort A mice experienced less severe colitis than those receiving feces from cohort B mice (Supplementary Figure S1). Thus, the differences in sensitivity toward gut epithelial injury-induced colitis in these two cohorts were indeed related to their microbiota.

Pretreatment with *B. longum* JDM 301 on colitic mice resulted in improvement of body weight loss and recovery of colon length in cohort A mice compared to *B. longum* JDM 301 non-treated colitic mice. However, mice in cohort B did not show obvious sign of colitis improvement with *B. longum* JDM 301 pretreatment (Figures 1C,D). Microscopically, colonic epithelial damage and inflammatory cell infiltration were reduced in cohort A mice that were pretreated with *B. longum* JDM 301, while severe epithelial damage and inflammation remained in cohort B mice (Figures 1E,F). Furthermore, all the mice in cohort A with probiotic therapy survived the wasting disease; while 9 out of 9 mice in cohort B with probiotic pretreatment failed to survive within the experimental period. In aggregate, these data indicate that the effectiveness of the probiotic *B. longum* JDM 301is correlated with host microbiota.

### The Effectiveness of the Probiotic *B. longum* JDM 301 in Colitis Is Correlated With Neither Disease Severity nor a High-Fat Diet

The effectiveness of *B. longum* JDM 301 in relieving colitis in cohort A but not cohort B mice could be due to milder colitis in cohort A relative to cohort B mice. To rule out this possibility, we used a HFD to exacerbate colitis. Consumption of HFD is considered as one of the risk factors of IBD and HFD can exacerbate DSS-induced colitis in animals (Park et al., 2012; Cheng et al., 2016). We, therefore, fed 5- to 6-week-old C57Bl/6 mice from cohort A and B with HFD.

**FIGURE 2:**
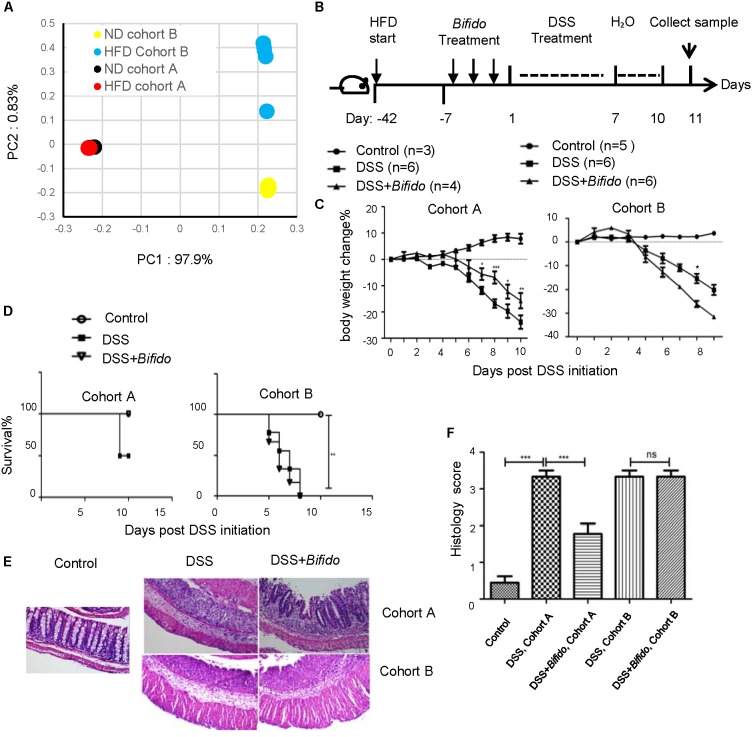
The effectiveness of the probiotic *B. longum* JDM 301 is not related to colitis severity or high-fat diet. **(A)** PCoA analysis illustrating the presence of different microbial community between HFD-fed mice from cohort **(A**,**B)**. Each symbol represents one individual mouse. The HFD fecal bacterial samples were collected 6 weeks after HFD and before probiotic and DSS treatment. **(B)** Schematic diagram for experimental design. **(C)** Body weight changes during DSS treatment in indicated mice. **(D)** Survival curve. **(E)** Representative images of H&E stained distal colon tissues from indicated mice (magnification: 200×). **(F)** Histologic scores. All data are given as means ± SEMs. ^∗^*P* < 0.05; ^∗∗^*P* < 0.01; ^∗∗∗^*P* < 0.001. The number of mice per group was 3∼6.

We first checked the similarity in microbiota composition in the two cohorts of mice fed with HFD. Six weeks after HFD, fecal pellets were collected, bacterial DNA was extracted and 16S rRNA gene sequences were analyzed. Based upon PCoA and Bray-Curtis distances analyses, HFD changed the microbiota landscape dramatically in cohort B, while it had limited impact on the microbial communities in cohort A mice (Figure 2A and Supplementary Figure S2). However, the microbiota differences between cohort A and B mice still existed (Figure 2A).

We then challenged the HFD-fed mice with DSS (Figure 2B). Consistent with the previous report (Thaiss et al., 2018), HFD exacerbated colitis especially in cohort A mice. The body weight of HFD-fed cohort A mice was reduced by more than 25% on day 10 after the DSS challenge (Figure 2C). Half of the HFD-fed mice in cohort A died after the DSS challenge (Figure 2D). On HFD-feeding, the intestinal inflammation and epithelial damage in cohort A mice became similar to that in cohort B mice (Figures 2E,F).

To examine whether the probiotics still had effect in this more severe form of colitis condition, the HFD-fed mice were pretreated with *B. longum* JDM 301 1 week before DSS challenge. Our data indicated that *B. longum* JDM 301 pretreatment still tempered DSS-induced colitis in HFD-fed cohort A mice, as it did in the ND condition. The body weight loss after DSS challenge was significantly relieved in mice from cohort A when pretreated with *B. longum* JDM 301 (Figure 2C). All the mice from cohort A receiving *B. longum* JDM 301 pretreatment survived, while half of those not receiving *B. longum* JDM 301 pretreatment died (Figure 2D). Also, the colonic inflammation became less severe on pretreatment with *B. longum* JDM 301 (Figures 2E,F). Hence, these results indicate that although HFD exacerbated colitis in cohort A mice, the probiotic effectiveness still remained.

In contrast to its effectiveness in cohort A mice, *B. longum* JDM 301 still failed to relieve IBD in cohort B mice fed on HFD and even promoted body weight loss (Figure 2C). There was still 100% mortality even after probiotic therapy (Figure 2D). There were no signs of improvement in epithelial integrity and colonic inflammation (Figures 2E,F). Altogether, the data indicated that the effectiveness of the probiotic *B. longum* JDM 301 in colitis was not correlated with either disease severity or HFD.

### Ecological Characteristics of the Gut Microbiota That Are Correlated With *B. longum* JDM 301 Efficacy

**FIGURE 3:**
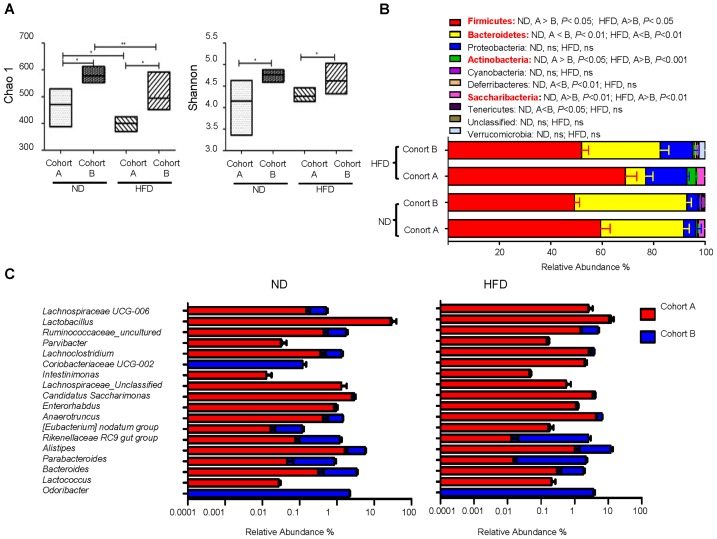
Ecological characteristics of the gut microbiota that are correlated with *B. longum* JDM 301 efficacy. The fecal bacterial samples were collected before probiotic and DSS treatment and bacterial 16S rRNA sequence data were analyzed accordingly. **(A)** α-diversity indicated by Chao1 (species richness) and Shannon index (species evenness). The line drawn in the middle of the box represents the median value and the box represents the range of values. **(B)** Taxonomic composition at the phyla level in the indicated mice. **(C)** Taxonomic composition at genera level in the indicated mice under ND and HFD. The top 18 genera that were significantly different (*P* < 0.05) between the two cohorts in both ND and HFD conditions were shown. ^∗^*P* < 0.05; ^∗∗^*P* < 0.01.

To look for the ecological characteristics of the gut microbiota that are correlated with probiotic effectiveness, we compared the overall microbial community configurations in probiotic-sensitive cohort A and probiotic-insensitive cohort B mice at both ND- and HFD-feeding conditions. The comparison was done at the baseline level, i.e., before pretreatment with *B. longum* JDM 301and challenge with DSS. Significant differences in both species richness represented by Chao1 index and species evenness represented by Shannon’s index were observed between cohort A and cohort B mice (Figure 3A). Both indices were bigger in cohort B mice compared to those in cohort A mice, irrespective of the diet used. HFD-feeding reduced total species richness (Chao1) in both cohorts, but species evenness (Shannon index) was not disturbed by HFD (Figure 3A). At the phyla level, cohort A mice had more Firmicutes, Actinobacteria, Saccharibacteria and less Bacteroidetes compared to cohort B at both ND- or HFD-feeding conditions (Figure 3B). Shifting diet from ND to HFD resulted in an increase in phylum Proteobacteria in both cohorts (from 1 and 5% to 14%, respectively) (Figure 3B). This is consistent with the notion that Proteobacteria expansion is an indicator of colon epithelial dysfunction and correlates to the increase of sensitivity to DSS-induced colitis at HFD condition (Litvak et al., 2017). However, Proteobacteria was not a good indicator for the *B. longum* JDM 301’s efficacy, as the relative abundance of Proteobacteria were not different between cohort A and B at both ND- or HFD-feeding conditions (Figure 3B). Furthermore, the relative abundance of 18 specific genera varied consistently between the two cohorts of mice irrespective of the diet used (Figure 3C). Among them, *Alistipes* and *Parabacteroides*, two genera that have been implied participating in IBD pathogenesis (Feng et al., 2015), were increased significantly in cohort B compared with those in cohort A mice fed with either ND or HFD. How these 18 genera might be related to *B. longum* JDM 301’s efficacy need further investigation.

### *B. longum* JDM 301 Has Limited Ability to Change the Taxonomic Composition of the Gut Microbiota

**FIGURE 4:**
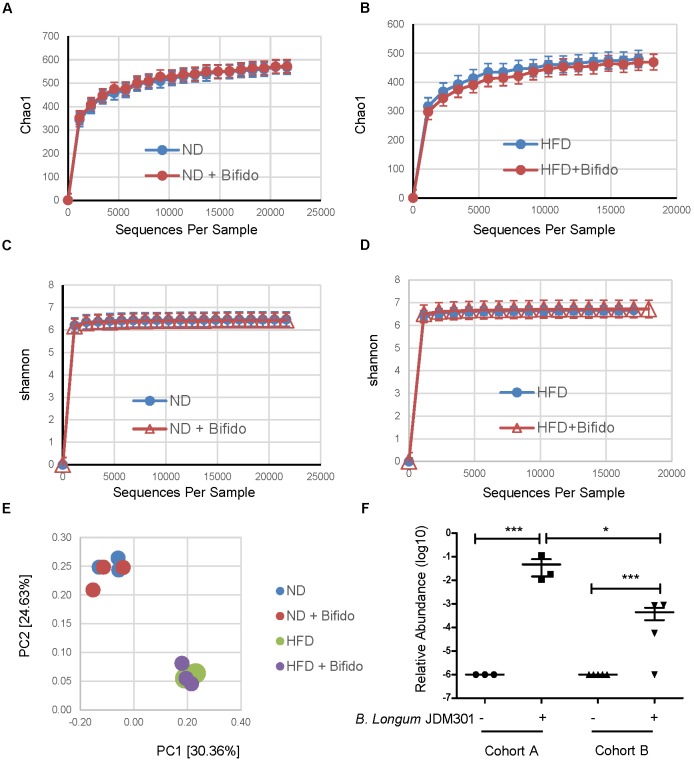
*B. longum* JDM 301 has limited ability to change the taxonomic composition of the gut microbiota. **(A–D)** High-throughput sequencing of 16S rRNA in fecal bacterial DNA from WT mice in batch A fed with ND or HFD before or 6 days after *B. longum* JDM 301 treatment. Chao1, indicative of bacterial species richness (a and b), Shannon, indicative of bacterial species evenness **(C**,**D)**. **(E)** PCoA analysis of the microbiota composition in indicated mice. Each symbol represents one individual mouse. **(F)** The relative abundance of fecal *Bifidobacterium* spp. in indicated mice before or 6 days after *B. longum* JDM 301 treatment. ^∗^*P* < 0.05; ^∗∗∗^*P* < 0.001.

The mechanism behind different probiotic efficacy in colitis in the same strain of mice with different microbiota is not clear. We first questioned how pretreatment with *B. longum* JDM 301 effectively alleviated colitis in cohort A mice. One possibility is that *B. longum* JDM 301 might be able to change the host microbiota so that the harmful members of microbes participating in IBD declined in cohort A mice. To test this, we collected feces from *B. longum* JDM 301 untreated and treated cohort A mice one day prior to DSS challenge and then performed high-throughput gene-sequencing analysis of fecal bacterial16S rRNA. We used rarefaction analysis to compare bacterial diversity within individual mice of a group (α diversity) in both ND- and HFD-fed conditions. *B. longum* JDM 301 treatment did not change species richness (Chao1) (Figures 4A,B) and species evenness (Shannon index) significantly (Figures 4C,D). PCoA analysis of the microbiota composition in *B. longum* JDM 301-treated mice did not show a different community composition relative to that of *B. longum* JDM 301-untreated mice at both ND- and HFD-fed conditions (Figure 4E). Thus, the impact of *B. longum* JDM 301on the taxonomic composition of the fecal microbiota in cohort A mice was very limited even though it succeeded in the induction of colitis remission in those mice.

Another possible factor that can potentially influence the probiotic effectiveness is related to its colonization abilities in the host gut. To determine whether oral delivery of *B. longum* JDM 301 could result in its engraftment in the gut, we checked fecal *Bifidobacterium* spp. enrichment 6 days after *B. longum* JDM 301 treatment and before DSS challenge in both cohorts of mice under ND condition via 16S rRNA gene sequence data analysis. The relative abundance of *Bifidobacterium* spp. was increased in both cohorts 6 days after *B. longum* JDM 301 treatment, however, the amplitude of increment was bigger in cohort A than that in cohort B mice (Figure 4F). The varied probiotic engraftment efficacy could at least partially explain the different efficacies of the probiotic *B. longum* JDM 301 in colitis in these two cohorts of mice.

## Discussion

In this work, we measured the ability of gut microbiota and HFD to influence the host response to a model probiotic, *B. longum* JDM 301, in a DSS-induced mouse colitis model. We demonstrated that the probiotic effectiveness in preventing colitis can be varied across individual mice even when they have the same genetic background. We further showed different microbiome features were highly correlated with different probiotic response. Consumption of the diet rich in fat can change the host sensitivity to mucosal injury-induced colitis but may not necessarily change the host responsiveness to probiotic therapy. Thus, it is likely that the gut microbiota utilizes different mechanisms in regulating colitis development and modulating probiotic colonization and functions.

Our results of mice with the same genetic background but different microbiota having different sensitivity to colitis induction are very similar to a previous report, which showed that C57Bl/6 from the UC Berkeley and JAX colony had different intestinal microbiota and had also significantly different sensitivity to DSS-induced wasting diseases (Schieber et al., 2015). Furthermore, our data are also consistent with the notion that the intestinal microbiota contributes to the immunopathogenesis of IBD (Kaser et al., 2010).

Although probiotics are defined as beneficial microorganisms to the host, exact mechanisms of how probiotics function inside the host remain incompletely understood. Possible pathways that have been suggested for how probiotics works include: (1) restoring microbial imbalances, (2) enhancing the epithelial barrier function, and/or (3) modulating the immune responses (Abraham and Quigley, 2017). The bacterial species that can be called probiotics are still expanding (El Hage et al., 2017), but whether one type of probiotic fits for all people at the same or different disease conditions is currently not known. Our data suggested that the individual host gut microbiome can probably influence whether a given probiotic can have beneficial effects on the specific host or not.

How personalized microbiota influence probiotic effectiveness requires further investigation. One possible way is to influence the probiotic engraftment efficacy as our data and others have suggested (Stecher et al., 2010; Lee et al., 2013; Maldonado-Gomez et al., 2016). Another possible way is to influence probiotic’s functions. For example, different microbiota might have different ability to influence probiotics in generating short-chain fatty acids, which are microbiota-derived metabolites that play important roles in mucosal protection and wound healing (van der Beek et al., 2017). Furthermore, one earlier study indicated that when certain gut microbes translocated to the internal tissue, they can induce disease tolerance (Schieber et al., 2015). Many more other possible mechanisms remain to be determined.

Our data further suggested that it might be possible to predict probiotic efficacy via analysis of the host microbial and genetic features. Personalized measurements including gut microbiome have been shown to be able to more accurately predict postprandial glycemic response for each unique person (Zeevi et al., 2015), they might also help for personalized probiotic therapies.

In aggregate, this study demonstrated a correlation between host microbiota and the probiotic therapeutic efficacy in colitis. Therefore, carefully monitoring personal and microbiome features might be needed for a successful probiotic biotherapy for clinical IBD patients.

## Author Contributions

YW and KZ conceived and designed the experiments. SS performed most of the experiments and contributed to the manuscript preparation. QW helped the probiotics preparation. WL, QL, HS, ML, JG, and BZ conducted histopathology assessment. YK, ZL, and YXW contributed to the high-throughput fecal bacterial 16S rRNA gene sequence analysis. YW and SS wrote the paper.

## Conflict of Interest Statement

The authors declare that the research was conducted in the absence of any commercial or financial relationships that could be construed as a potential conflict of interest.
