# Supplementary material for: The Probiotic Effectiveness in Preventing Experimental Colitis Is Correlated With Host Gut Microbiota
Source: Front Microbiol. 2018 Nov 1;9:2675. doi: 10.3389/fmicb.2018.02675 (PMC6223222; doi:10.3389/fmicb.2018.02675)
Supplement: Supplementary file 1 [file Data_Sheet_1.docx]

Supplementary Material

**The probiotic effectiveness in preventing experimental colitis is correlated with host gut microbiota**

**Sharmila Suwal, Qiong Wu, Wenli Liu, Qingya Liu, Hongxiang Sun, Ming Liang, Jing Gao, Bo Zhang, Yanbo Kou, Zhuanzhuan Liu, Yanxia Wei, Yugang Wang*, Kuiyang Zheng***

*** Correspondence:**Yugang Wang or Kuiyang Zheng
wang_yg@hotmail.com, or ZKY02@163.com

## Supplementary Figures


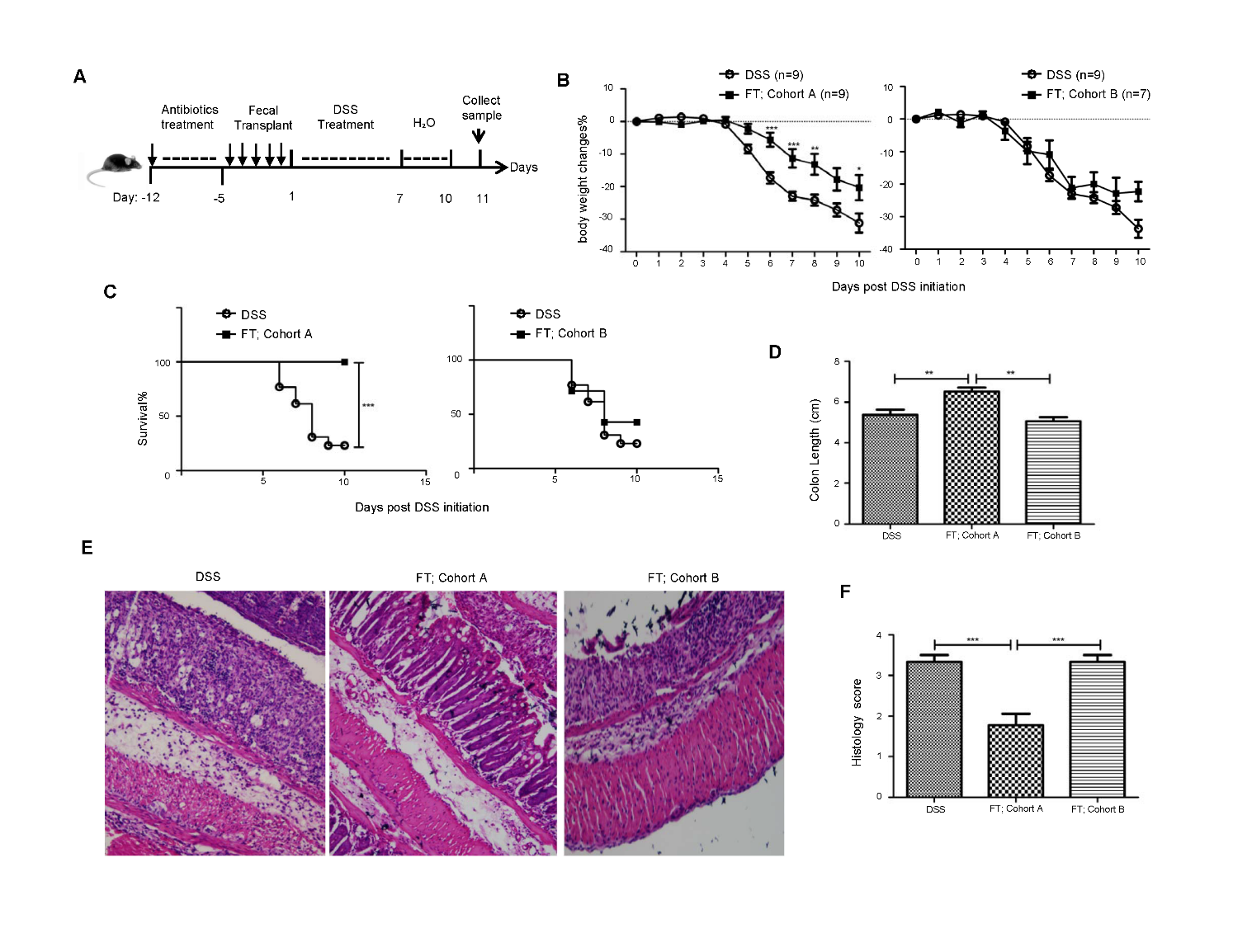


**Supplementary Figure S1.** Host commensal microbiota dramatically affect DSS-induced colitis severity. Fecal samples collected from cohort A and B mice were transferred to male C57Bl/6 WT mice. Mice challenged only with DSS but received no antibiotics and fecal transplantation were used as control (DSS group). **(a)** Schematic diagram of experimental design. **(b)** The body weight changes during DSS treatment. **(c)** Survival curve. **(d)** Mean colon length in cm. Colons were collected on day 10 post DSS initiation. **(e)** Representative images of H&E stained distal colon tissues from indicated mice (magnification: 200x). **(f)** Histologic scores. All data are given as means±SEMs. **P*<0.05, ***P*<0.01; ****P*<0.001. FT, fecal transplant.

**Supplementary Figure S2.**  HFD-feeding changed microbiota landscape in cohort B mice. Mice were fed on high-fat diet for 6 weeks. All Fecal samples were collected before DSS challenge. Bray-Curtis distances
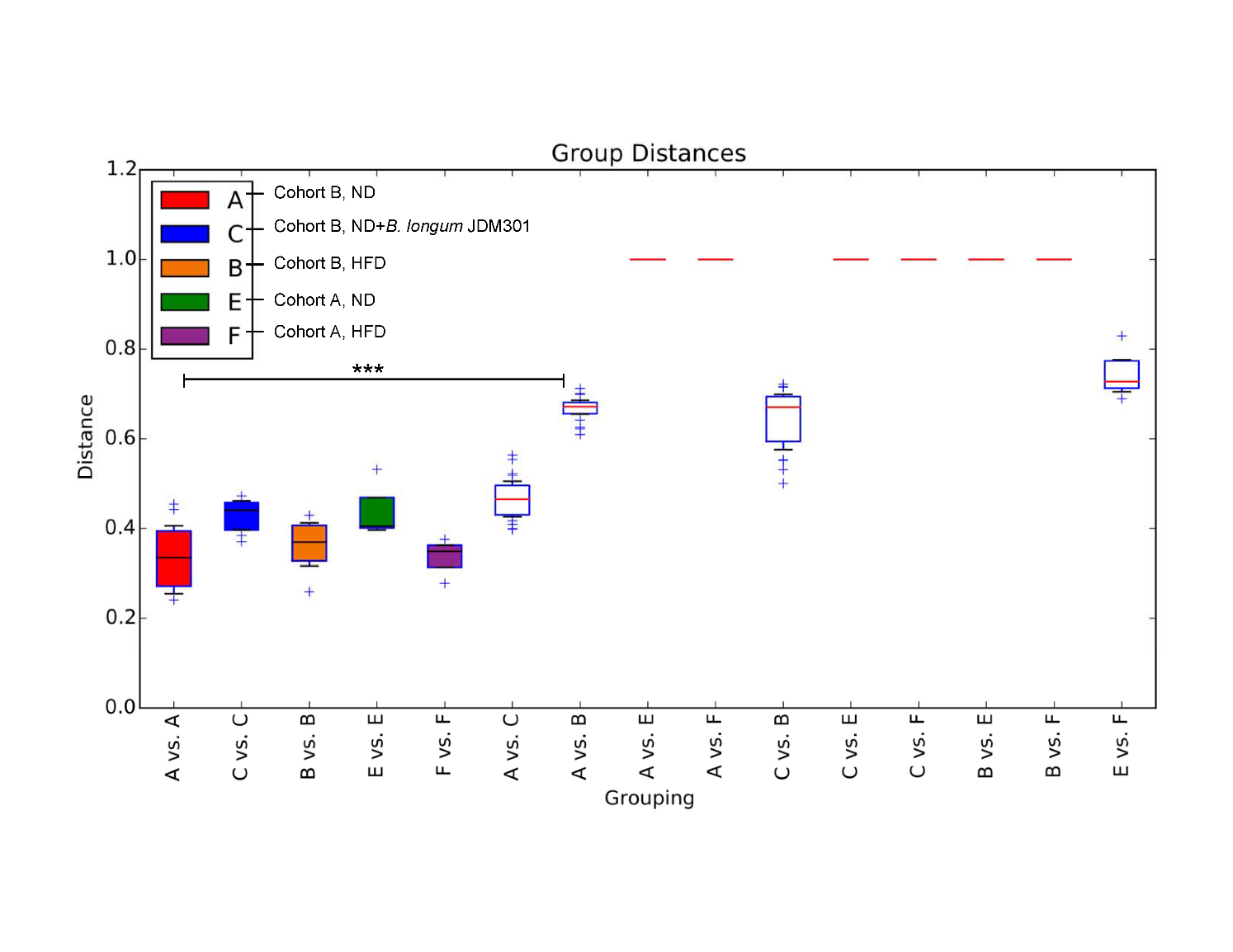
 between different groups of mice as indicated were calculated according to fecal bacterial 16S rRNA sequencing data. ****P*<0.001. ND, normal diet; HFD, high-fat diet.
